# Coordination of Division and Development Influences Complex Multicellular Behavior in *Agrobacterium tumefaciens*

**DOI:** 10.1371/journal.pone.0056682

**Published:** 2013-02-20

**Authors:** Jinwoo Kim, Jason E. Heindl, Clay Fuqua

**Affiliations:** Department of Biology, Indiana University, Bloomington, Indiana, United States of America; East Carolina University School of Medicine, United States of America

## Abstract

The α-Proteobacterium *Agrobacterium tumefaciens* has proteins homologous to known regulators that govern cell division and development in *Caulobacter crescentus*, many of which are also conserved among diverse α-Proteobacteria. In light of recent work demonstrating similarity between the division cycle of *C. crescentus* and that of *A. tumefaciens*, the functional conservation for this presumptive control pathway was examined. In *C. crescentus* the CtrA response regulator serves as the master regulator of cell cycle progression and cell division. CtrA activity is controlled by an integrated pair of multi-component phosphorelays: PleC/DivJ-DivK and CckA-ChpT-CtrA. Although several of the conserved orthologues appear to be essential in *A. tumefaciens*, deletions in *pleC* or *divK* were isolated and resulted in cell division defects, diminished swimming motility, and a decrease in biofilm formation. *A. tumefaciens* also has two additional *pleC/divJ* homologue sensor kinases called *pdhS1* and *pdhS2*, absent in *C. crescentus*. Deletion of *pdhS1* phenocopied the Δ*pleC* and Δ*divK* mutants. Cells lacking *pdhS2* morphologically resembled wild-type bacteria, but were decreased in swimming motility and elevated for biofilm formation, suggesting that *pdhS2* may serve to regulate the motile to non-motile switch in *A. tumefaciens.* Genetic analysis suggests that the PleC/DivJ-DivK and CckA-ChpT-CtrA phosphorelays in *A. tumefaciens* are vertically-integrated, as in *C. crescentus*. A gain-of-function mutation in CckA (Y674D) was identified as a spontaneous suppressor of the Δ*pleC* motility phenotype. Thus, although the core architecture of the *A. tumefaciens* pathway resembles that of *C. crescentus* there are specific differences including additional regulators, divergent pathway architecture, and distinct target functions.

## Introduction

Developmental and morphological asymmetry is critical to the lifestyle of both eukaryotes and prokaryotes. Work in several experimental systems has begun to detail, at the molecular level, examples of cellular asymmetries among both Gram negative and Gram positive bacteria [1], [2]. Bacterial cell division provides one striking example [3]. For bacteria that divide by binary fission, such as *Escherichia coli* and *Bacillus subtilis*, each division cycle generates two distinct poles of these rod-shaped bacteria. The ‘old’ pole is inherited from the mother cell while the ‘new’ pole arises from the FtsZ-generated division septum. Related to this asymmetry, in many bacteria there is preferential localization of certain structures to a single pole, including flagella, pili, and secretion systems [4]. Importantly, the proper assembly and distribution of these polar functionalities are critical for these bacteria to interact with one another and with their environment.

**Figure 1:**
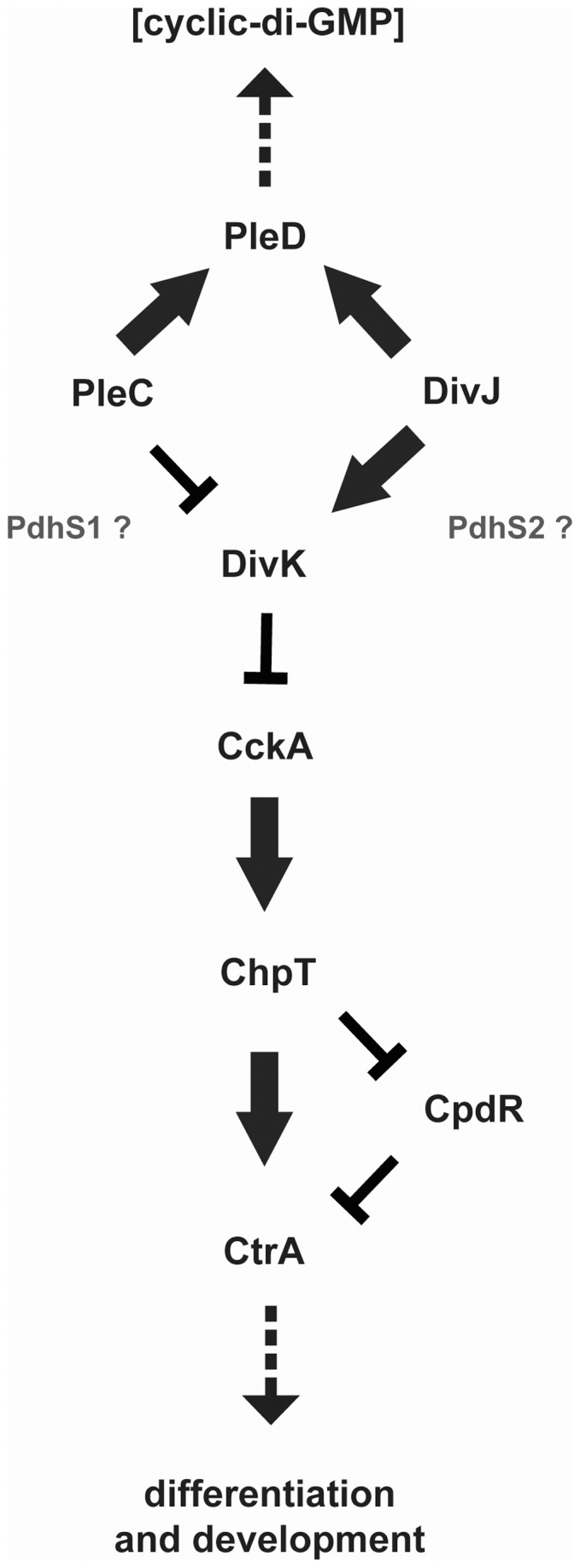
Coordination of division and development (CDD) regulatory pathway for *Caulobacter crescentus*. Black protein names indicate known proteins in *C. crescentus* for which homologues are present in *A. tumefaciens* and many other α-Proteobacteria. Grey protein names indicate proteins found in *A. tumefaciens* but not *C. crescentus* and which likely participate in the CDD regulatory pathway in *A. tumefaciens*. Arrows signify positive regulatory interactions while bars indicate negative regulatory interactions. Dashed arrows indicate observed output phenotypes. The position of PdhS1 and PdhS2 along the pathway is hypothetical.

Prokaryotic developmental and morphological asymmetry has been intensively studied in *Caulobacter crescentus*, an overtly asymmetric, prosthecate member of the α-Proteobacteria [5], [6]. *C. crescentus* has a biphasic mode of growth in which it divides to produce two non-identical daughter cells: a sessile stalked cell and a motile swarmer cell. In *C. crescentus,* the transcription factor CtrA is the master regulator of cell cycle progression and its activity is exquisitely regulated through two vertically integrated phosphorelays [7], [8] (Fig. 1). The current model for CtrA control is that a bifurcated phosphorelay leads from the hybrid histidine kinase CckA through the histidine phosphotransferase ChpT that may phosphorylate either CtrA or a second response regulator CpdR [9]. Phospho-CtrA is active for DNA binding and directly controls expression of key cell-cycle genes and inhibits DNA replication by binding to the origin of replication. Unphosphorylated CpdR is thought to stimulate proteolytic turnover of CtrA, whereas phospho-CpdR is inactive. Active CckA thus results in phosphorylation of CtrA and CpdR, simultaneously activating and stabilizing CtrA. CckA is itself regulated by the response regulator DivK, which in its phosphorylated form inhibits CckA autophosphorylation by interrupting the interaction between CckA and the unusual kinase DivL [10]. DivK phosphorylation, and its inhibitory activity on CckA, is controlled by two parallel histidine kinases, PleC and DivJ [11]–[13]. The localization and activity of PleC and DivJ are opposed in late predivisional cells such that DivK activity remains high in the stalked cell compartment and low in the swarmer cell compartment. Following cell division, this results, ultimately, in high CtrA activity in the swarmer cell and decreased CtrA activity in the stalked cell. In addition to their interaction with DivK both PleC and DivJ influence the activity of a second response regulator PleD that leads to alterations in levels of the intracellular second messenger bis-(3′–5′)-cyclic dimeric guanosine monophosphate (c-di-GMP) [13]–[15]. One consequence of the resulting increase in c-di-GMP levels is the PopA-dependent degradation of CtrA [16].

The individual components of the PleC/DivJ-DivK/PleD and CckA-ChpT-CtrA/CpdR phosphorelays (designated here as the CDD regulatory pathway, for coordination of division and development) are highly conserved among many α-Proteobacteria [17]. Additional PleC-DivJ homologue sensor kinases (PdhS-type) are present within the Rhizobiales and Rhodobacterales [18]. PdhS-type homologues appear primarily within host-associated (commensal and pathogenic) bacteria, perhaps indicating their participation in regulating adaptation to the host environment in these organisms. These include PdhS in the animal pathogen *Brucella abortus* and CbrA in the legume symbiont *Sinorhizobium meliloti* [19], [20]. PdhS-type homologues, aside from PleC and DivJ, are notably absent from the Caulobacterales, including *C. crescentus*. How these putative two-component sensor kinases are integrated into the CDD regulatory pathway has not been well studied.

*Agrobacterium tumefaciens* is a free-living, soil-dwelling, rod-shaped member of the α-Proteobacteria [21]. *A. tumefaciens* is best known for its ability to stably transfer a specific segment of its DNA (the T-DNA) to plants and, when appropriately engineered, to fungi and mammalian cells [22]–[24]. *A. tumefaciens* is a common member of the soil microbiome and interacts with plant roots in multicellular associations called biofilms. Initial association of this bacterium with surfaces occurs at a single pole of the bacterium where a newly identified adhesin is localized [25]–[27]. *A. tumefaciens* has been extensively used as a model organism for the study of host-pathogen interactions, type IV secretion (T4S), quorum sensing, and biofilm formation [28]–[31]. Furthermore, cell division in *A. tumefaciens* occurs via a recently described polar budding process rather than the canonical binary fission found in many other rod-shaped bacteria [32]. We report here initial observations in *A. tumefaciens* analyzing the architecture of the CDD regulatory pathway and linking these phosphorelay components to its developmental program. We also describe results indicating that two PdhS-type homologues, PdhS1 and PdhS2, can influence cell division and development in *A. tumefaciens*.

## Materials and Methods

### Strains, Plasmids, and Growth Conditions

Bacterial strains and plasmids used in this study are listed in Table S1. *A. tumefaciens* C58 was routinely cultivated at 28°C in AT minimal medium plus 1% (w/v) glucose as a carbon source and 15 mM (NH_4_)_2_SO_4_ as a nitrogen source (ATGN), without exogenous FeSO_4_ [33]–[35]. For biofilm assays 22 µM FeSO_4_ was included in the media. *E. coli* DH5α λ *pir*, TOP10 F′, and S17-1 λ *pir* were routinely cultivated at 37°C in lysogeny broth (LB) [36]–[38]. Antibiotics were used at the following concentrations (*A. tumefaciens*/*E. coli*): Ap, ampicillin (100/50 µg·mL^−1^), Km, kanamycin (150/25 µg·mL^−1^), and Gm, gentamicin (150/30 µg·mL^−1^). Measurements of *A. tumefaciens* growth rates were performed by inoculating 5 mL of ATGN to a final optical density at 600 nm (OD_600 nm_) = 0.012 and incubating at 28°C with aeration. Calculation of growth rates and doubling times used a publicly accessible doubling time calculator (http://www.doubling-time.com/compute.php). Viable cell counts were determined by plating serial dilutions of freshly inoculated cultures onto ATGN agar plates and incubating for three days at 28°C.

### Generation of Nonpolar, Markerless Deletions

Nonpolar, markerless deletions of the *A. tumefaciens divJ* (Atu0921), *pleC* (Atu0982), *pdhS1* (Atu0614), *pdhS2* (Atu1888), *divK* (Atu1296), and *pleD* (Atu1297) homologues were generated using splicing by overlap extension (SOE) polymerase chain reaction (PCR) [39]. Oligonucleotides used in this study are listed in Table S2. A fragment extending 500-bp upstream of the start codon was amplified using primers “P1” and “P2”. A downstream 500-bp fragment beginning at or near the stop codon was amplified using primers “P3” and “P4”. P2 and P3 for each gene were designed to contain roughly 21 bp of homology (the “overlap”). Amplified fragments were gel purified and used as template along with primers P1 and P4 in a second round of PCR to generate an approximately 1 kb SOE fragment containing a fusion of the upstream and downstream regions of the targeted locus. This SOE amplicon was ligated with plasmid pGEM-T Easy (Promega) and sequenced. Individual SOE fragments were sub-cloned into the pNPTS138 suicide vector carrying kanamycin resistance and *E. coli sacB*-mediated sucrose sensitivity. pNPTS138 is a ColE1 plasmid and as such is unable to replicate in *A. tumefaciens*. Transfer of the pNPTS138-derived suicide plasmid into *A. tumefaciens* was achieved with biparental mating from the *E. coli* donor strain S17-1 λ *pir* [38]. One mL each from overnight cultures of the donor strain and *A. tumefaciens* recipient were pelleted and resuspended in 50 µL LB, mixed, and spotted onto a nitrocellulose filter on the surface of an LB agar plate. After overnight growth at 28°C the mating pool was resuspended in 1 mL ATGN and serial dilutions were plated onto ATGN agar plates supplemented with 300 µg·mL^−1^ Km, selecting for *A. tumefaciens* cells that had integrated the pNPTS138 derivative by homologous recombination. The *E. coli* donor strain S17-1 λ *pir* is a proline auxotroph and is unable to grow on ATGN. Following three days incubation at 28°C colonies were restreaked onto both ATGN agar plates containing 300 µg·mL^−1^ Km and ATSN agar plates (ATGN with 5% sucrose and no glucose). Km resistance combined with sucrose sensitivity (Suc^S^) indicates that a candidate transconjugant has integrated the pNPTS138 derivative into the genome at the targeted locus by single crossover. Recombinant candidates were then grown overnight at 28°C in ATGN in the absence of Km and plated the following day onto ATSN agar plates to select for sucrose resistant (Suc^R^) allelic replacement candidates. After three days growth at 28°C colonies were patched in parallel onto ATGN Km and ATSN plates. Km^S^ Suc^R^ recombinants were then tested for the targeted deletion by diagnostic PCR using primers external to the locus (labeled P5 and P6 in Table S2) as well as internal primers. Candidate colonies were further streak purified and verified a second time by diagnostic PCR before being used in subsequent assays.

### Cloning of Sensor Kinase and Response Regulator Genes

To generate complementation constructs the coding sequence for each gene was amplified via PCR with Phusion High-Fidelity DNA polymerase (Thermo Fisher) using purified wild-type *A. tumefaciens* C58 genomic DNA as template and the indicated primers (Table S2). Amplicons were gel purified using the E.Z.N.A. gel extraction kit per instructions provided by the manufacturer (Omega Bio-Tek), ligated into pGEM-T Easy, and sequenced. Coding sequences were sub-cloned into the broad-host range vectors pSRKKm and pSRKGm [40], bearing Km^R^ and Gm^R^, respectively, using the following restriction endonucleases (New England Biolabs): *pleC* and *pdhS1* (XbaI and XhoI), *pdhS2* (NdeI and NheI), *divK* (NdeI and XbaI), and *pleD* (NdeI and BamHI). Successful subcloning was confirmed by sequencing. Plasmid clones were maintained in *E. coli* and purified using the E.Z.N.A. plasmid mini kit I per instructions provided by the manufacturer (Omega Bio-Tek). Constructs were transformed into electrocompetent *A. tumefaciens* by electroporation at 1.8 kV using the *E. coli* Pulser Transformation Apparatus (Bio-Rad) [41].

### Genomic Resequencing

Spontaneous suppressors of the Δ*pleC* swimming motility phenotype were isolated from 0.3% swim agar plates following incubation at room temperature for seven days. Bacteria isolated from the edge of motile flares were streak purified twice on ATGN. Total genomic DNA from one of these suppressor strains was used to generate a paired-end library following the modified protocol of Lazinski and Camilli (http://genomics.med.tufts.edu/home/sampleprep). Approximately 20 µg sheared genomic DNA was blunt-ended using the NEB Quick Blunting Kit (New England Biolabs). Following blunt-ending the large fragment of DNA Polymerase I (Klenow fragment) was used to add one deoxyadenosine to the 3′ ends of the DNA preparation. This DNA preparation was ligated with an adapter mix consisting of primers OLJ131 and OLJ137 using the NEB Quick Ligation Kit (New England Biolabs). Finally, library amplification was performed by PCR with primers OLJ139 and OLJ140. Sequencing was performed on an Illumina HiSeq 2000 at the Tufts University Core Facility.

### Evaluation of Cell Morphology and Microscopic Localization of FtsZ

Evaluation of cell morphology and localization of an FtsZ-green fluorescent protein (GFP) fusion protein for each strain was performed on a Nikon E800 fluorescence microscope equipped with a Photometrics Cascade cooled CCD camera. For FtsZ-GFP localization plasmid pJW164G carrying an IPTG-inducible C-terminal GFP translational fusion to the primary *A. tumefaciens* FtsZ homologue (Atu2086) was transformed by electroporation into the indicated strains [32], [42]. Overnight cultures of pJW164G-transformed strains in ATGN plus appropriate antibiotic were back-diluted the morning of observation into fresh ATGN plus appropriate antibiotic and 100 µM IPTG. Cultures were grown at 28°C with aeration until ∼OD_600 nm_ = 0.5–0.8. 3 µL of the culture were placed on a 1% agarose pad on a clean glass slide and a clean 22×22 mm number 1.5 glass coverslip placed on top. Images were acquired using a 100X oil immersion objective and phase contrast optics or epifluorescence with a FITC-HYQ filter set (Nikon; excitation filter = 480/40 nm, dichromatic mirror = 505 nm, absorption filter = 535/50 nm). Cell morphology was not altered by expression of pJW164G and ectopic expression of GFP alone did not affect cell morphology and resulted in diffuse fluorescence in all strains (data not shown) [43]. Quantification of cell morphology and FtsZ-GFP localization patterns was performed on three independent blinded samples for each strain, with at least 19 individual cells counted per replicate and a total of at least 100 cells counted per strain.

### Evaluation of Swimming Motility and Flagellar Localization

Swimming motility of individual strains was evaluated both microscopically and on semisolid media [44]. Bright field microscopy on exponentially growing cultures was performed using a Zeiss Axioskop 40 equipped with an AxioCam MRm monochrome digital camera. Swim plates containing 0.3% agarose in ATGN, supplemented with 1 mM IPTG when appropriate, were inoculated with a single colony of the indicated strain at a central point and incubated for 7 days at 28°C. Swim ring diameters were recorded daily, however only the final swim ring diameters (day seven) are reported. Qualitative staining of flagella used a two-component stain modified from Mayfield and Inniss [45]. Component A contained equal volumes of 5% phenol and saturated AlK(SO_4_)_2_·12H_2_O in 10% tannic acid. Component B contained 12% crystal violet in 100% ethanol. Working staining solution was generated by combining component A and component B in a 10∶1 ratio, vortexing to mix, and centrifuging (2 minutes, 13,000×*g*) to remove precipitate. Ten µL of staining solution was applied to a wet mount of three µL of exponential phase culture and the slide observed within 5 min of staining.

### Static Culture Biofilm Formation

The ability of each mutant to form a biofilm was quantified using plastic coverslips in static cultures [43], [44]. Overnight cultures were back-diluted in fresh ATGN to OD_600 nm_ = 0.1 and grown with aeration at 28°C until OD_600 nm_ = 0.25–0.6. Cultures were diluted to OD_600 nm_ = 0.05 and 3 mL were added to each of four wells in a 12-well plate. A single coverslip was placed vertically into each well to submerge approximately half of each coverslip. Plates were incubated undisturbed at 28°C for 48 h. Coverslips were removed from each well, rinsed with water, and adherent biomass stained by 5 min immersion in a 0.1% (w/v) crystal violet solution. Stained coverslips were rinsed three times with water and allowed to air dry. Adsorbed crystal violet was solubilized by immersion in 200 µL 33% acetic acid and the absorbance of this solution determined at 600 nm (A_600 nm_) on a Synergy HT multi-detection microplate reader (Bio-Tek). Culture density for each sample was also determined by measuring the OD_600 nm_ of 200 µL of each culture. Data are typically presented as A_600 nm_/OD_600 nm_ ratios normalized to values obtained for the wild-type strain within each experiment. Each mutant was evaluated in three independent experiments each of which contained three technical replicates.

### Extracellular Polysaccharide Staining

Differential production of extracellular polysaccharides was examined by inoculating 5 µL of a turbid overnight culture of the indicated strains onto ATGN agar plates containing either 100 µg·mL^−1^ Congo Red (Sigma-Aldrich) or 200 µg·mL^−1^ Calcofluor White (Sigma-Aldrich) [46], [47]. Plates were incubated in the dark for three days at 28°C. Images for Congo Red plates were acquired using a Sony DSC-F828 digital camera under white light illumination. Images for Calcofluor White plates were acquired using a MultiImage Light Cabinet with ultraviolet illumination (Alpha Innotech Corporation).

### Short-term Binding Assay and Unipolar Polysaccharide Staining

Visualization of the unipolar polysaccharide (UPP) was achieved as previously described [27] by inoculating 3 µL of an exponential phase culture of each strain onto a glass slide-borne agarose pad containing 1.5% low-melting temperature agarose in water plus 0.01 µg·µL^−1^ Alexa Fluor 594-conjugated wheat germ agglutinin (fl-WGA, Invitrogen). A clean 22×22 mm number 1.5 glass coverslip was placed on top of the inoculum/agarose pad and lightly compressed to ensure even distribution. Slides were incubated in the dark for two hours at 28°C prior to observation under phase contrast and epifluorescence microscopy using the Nikon E800 setup described above with a G-2B filter set (excitation filter = 510–560 nm, dichromatic mirror = 575 nm, absorption filter = 610 nm). Fields were randomly chosen using phase contrast prior to imaging the lectin-stained UPP with fluorescent illumination.

Short-term binding assays evaluated the ability of individual strains to stably attach to a glass coverslip after one hour [44], [48]. Overnight cultures of each strain were back-diluted into fresh ATGN and aerated at 28°C. Exponential phase cultures were concentrated to a final OD_600 nm_ = 0.6 and two mL were placed on top of a glass coverslip in a six-well plate and incubated one h undisturbed at 28°C. Cultures were removed and coverslips washed 3× with ATGN, then inverted 20 min at room temperature on top of 100 µL ATGN containing 0.01 µg·µL^−1^ fl-WGA. Coverslips were finally washed once with ATGN containing 0.05% Tween-20 and three times with ATGN, then wet-mounted onto glass slides and observed as for UPP labeling above.

### Potato Disk Tumor Assay

The virulence potential of each strain was evaluated by monitoring the ability to initiate tumor formation on red potato slices [44], [49]. One cm diameter cores were obtained from organic red potatoes that had been washed in distilled water, incubated for 20 minutes in 10% bleach, and UV-irradiated for 20 min. Disks were sliced (0.5 cm) from the center portion of individual cores and placed onto 1.5% agar plates with no additional nutritional supplementation. Overnight cultures of the indicated strains were diluted to OD_600 nm_ = 0.6 in ATGN and serially diluted 10-fold and 100-fold. Fifty µL of each dilution were inoculated onto the potato surface and allowed to absorb. Plates were sealed with parafilm and incubated undisturbed for four weeks at room temperature. Tumors were enumerated on days 14, 21, and 28. Each strain was tested in three independent experiments containing five technical replicates per experiment per inoculum.

## Results

### Two Component Sensor Kinase Regulation of Development in *A. tumefaciens*

The *A. tumefaciens* genome contains four homologues of the *pleC*/*divJ* sensor histidine kinases from *C. crescentus*, all on the 2.84 Mb circular chromosome. In addition to genes that correspond to *pleC* and *divJ*, *A. tumefaciens* encodes two additional homologues, the PdhS-type kinases, *pdhS1* and *pdhS2*. To determine the role played by these genes in *A. tumefaciens* we attempted to create non-polar deletions of each open reading frame via allelic replacement. Deletions were readily generated for *pleC*, *pdhS1*, and *pdhS2*, indicating that none of these genes is essential. Deletion of the chromosomal *divJ* locus was only achieved when a second copy of the *divJ* coding sequence was provided *in trans* on a moderate-copy regulatable expression plasmid, suggesting that *divJ* is essential in this organism (data not shown).

**Figure 2:**
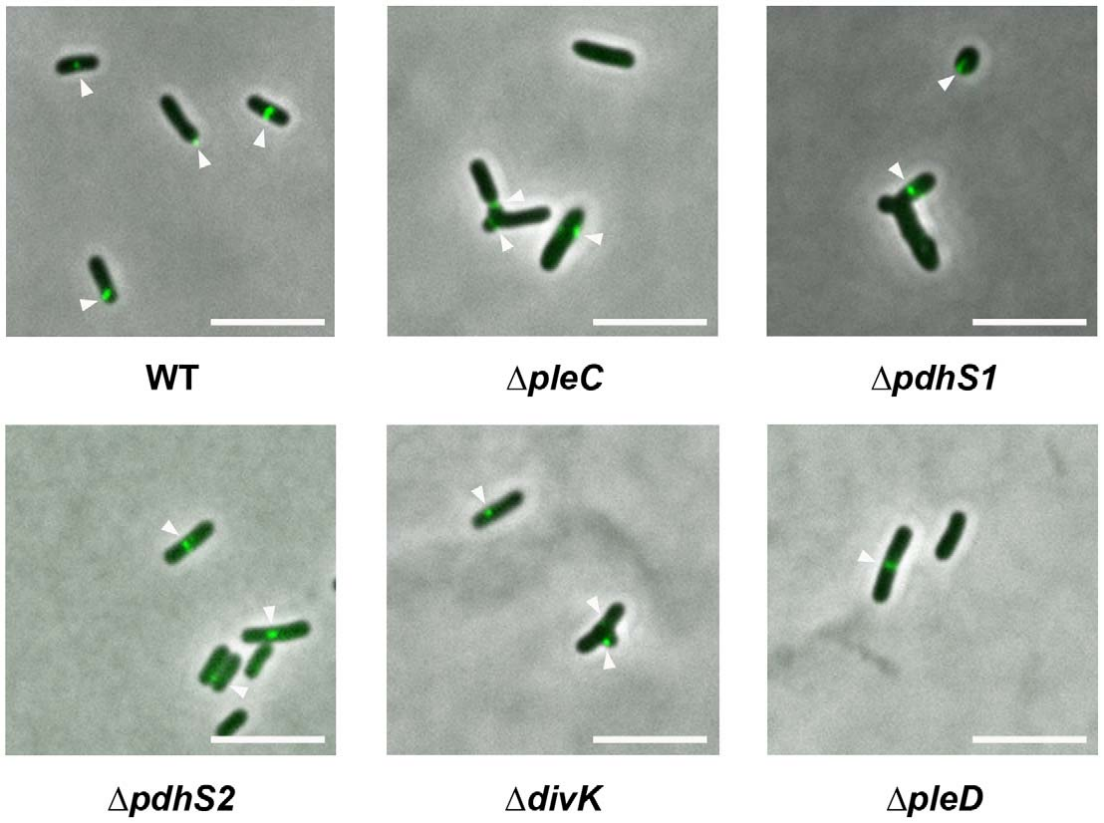
Localization of FtsZ and morphology of histidine kinase and response regulator mutants. Each strain carries a plasmid-borne copy of the *A. tumefaciens* FtsZ homolog (Atu2086) fused at its carboxy terminus to GFP and under transcriptional control of the *P_lac_* promoter. Expression was induced for 2–4 hours with 100 µM IPTG. 100X phase images were overlain with corresponding epifluorescence images showing FtsZ-GFP localization. Arrowheads indicate FtsZ-GFP localization. Note the aberrant morphology of the Δ*pleC*, Δ*pdhS1*, and Δ*divK* mutant strains. Scale bar = 5 µm.

The Δ*pleC*, Δ*pdhS1*, and Δ*pdhS2* mutant strains formed smaller colonies relative to wild-type *A. tumefaciens* on ATGN minimal medium plates, although their planktonic growth rates were not significantly slower than wildtype (Table 1). Despite apparently normal overall growth rates, both the Δ*pleC* and Δ*pdhS1* mutants exhibited distinctly aberrant cell division patterns. Wild-type *A. tumefaciens* grows as short, roughly symmetrical rods (Fig. 2, Table S3). In contrast, the Δ*pleC* and Δ*pdhS1* mutant strains frequently grew as elongated rods with one or more branches extending from one pole (Fig. 2, Table S3). Cell division in *A. tumefaciens* proceeds through an asymmetric budding process during which the future site of cell division appears as a sub-polar constriction where FtsZ localizes [32]. Cell elongation occurs unidirectionally from the pole adjacent to this site until the constriction, and FtsZ, is roughly at mid-cell. Finally, septation occurs at the constriction site and immediately following cell division a small focus of FtsZ is visible at the pole of the newly divided daughter cell. Thus, in unsynchronized wild-type cells the normal pattern of FtsZ localization includes either a mid-cell or sub-polar ring as well as an occasional polar focus. We hypothesized that the aberrant morphology of the Δ*pleC* and Δ*pdhS1* mutants may be due to altered regulation of the cell division machinery. Expression of a GFP fusion to the presumptive primary *A. tumefaciens* FtsZ homologue (Atu2086, *ftsZ2-gfp*) in the Δ*pleC* and Δ*pdhS1* mutants revealed either inappropriate or diffuse localization of this protein when compared with the predominantly mid-cell, and transient unipolar, localization of the Z-ring in wild-type *A. tumefaciens* (Fig. 2, Table S3). FtsZ-GFP regularly localized to sites of branching in these mutants suggesting that these branches may represent aberrant budding sites. The growth characteristics, morphology, and FtsZ localization pattern in the Δ*pdhS2* mutant were similar to wildtype (Fig. 2, Table S3).

**Table 1:** Doubling time and growth rate of CDD mutants.

Strain	Doubling Time (hours ± S.E.)	Growth Rate (doublings·h^−1^ ± S.E.)
WT	1.96 ± 0.33	0.37 ± 0.06
Δ*pleC*	1.69 ± 0.12	0.41 ± 0.03
Δ*pdhS1*	2.18 ± 0.19	0.32 ± 0.03
Δ*pdhS2*	1.84 ± 0.60	0.43 ± 0.12
Δ*divK*	1.84 ± 0.46	0.42 ± 0.13
Δ*pleD*	2.07 ± 0.66	0.38 ± 0.11

### Motility of Sensor Kinase Mutants

**Figure 3:**
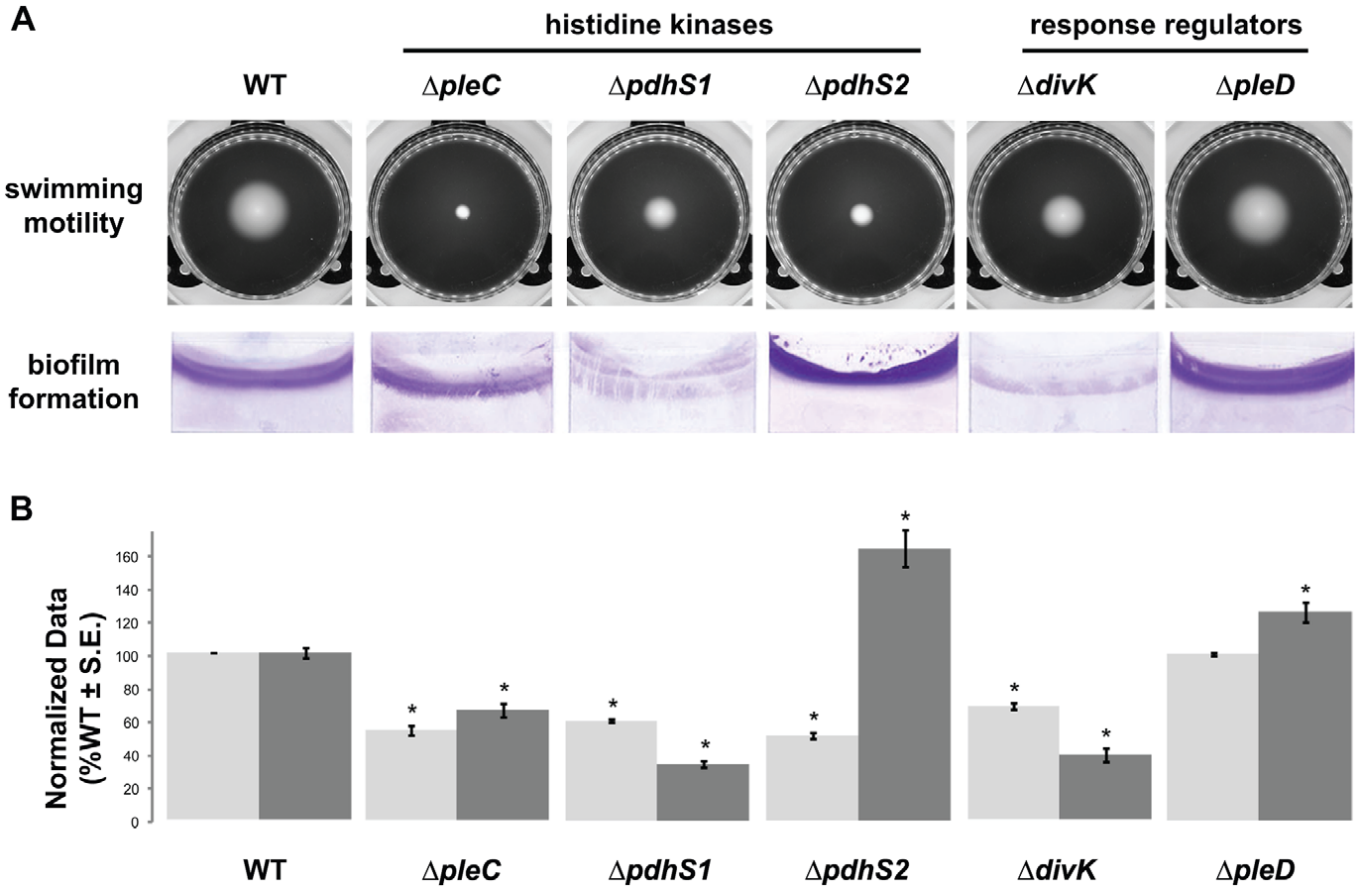
Biofilm formation and swimming motility of histidine kinase and response regulator mutants. (A) Swim ring formation after four days (top) and static biofilm formation after 48 h (bottom) for the indicated wild-type (WT) and mutant strains. (B) Quantification of adherent biomass (dark grey columns, right) and swim ring diameter (light grey columns, left) for the indicated wild-type (WT) and mutant strains. Swim ring diameters were measured after seven days growth in 0.3% swim agar plates at room temperature. Data for swim ring diameters are the mean of nine independent experiments (N = 9). Adherent biomass in 48 h biofilms on PVC coverslips was determined as solubilized crystal violet A_600_. In parallel the OD_600_ nm was measured for the planktonic bacteria in these cultures. Crystal violet absorbance was normalized to culture density by taking the ratio of A_600 nm_/OD_600 nm_. Data for biofilms are the mean of three independent experiments each of which contained three technical replicates (N = 3). For presentation all data were normalized to results obtained with the wild-type strain and are expressed as %WT ± S.E. Columns marked with an asterisk (*) indicate statistically significant differences between the mutant strain and the wild-type strain using Student’s *t* test (*P* ≤ 0.05).

**Figure 4:**
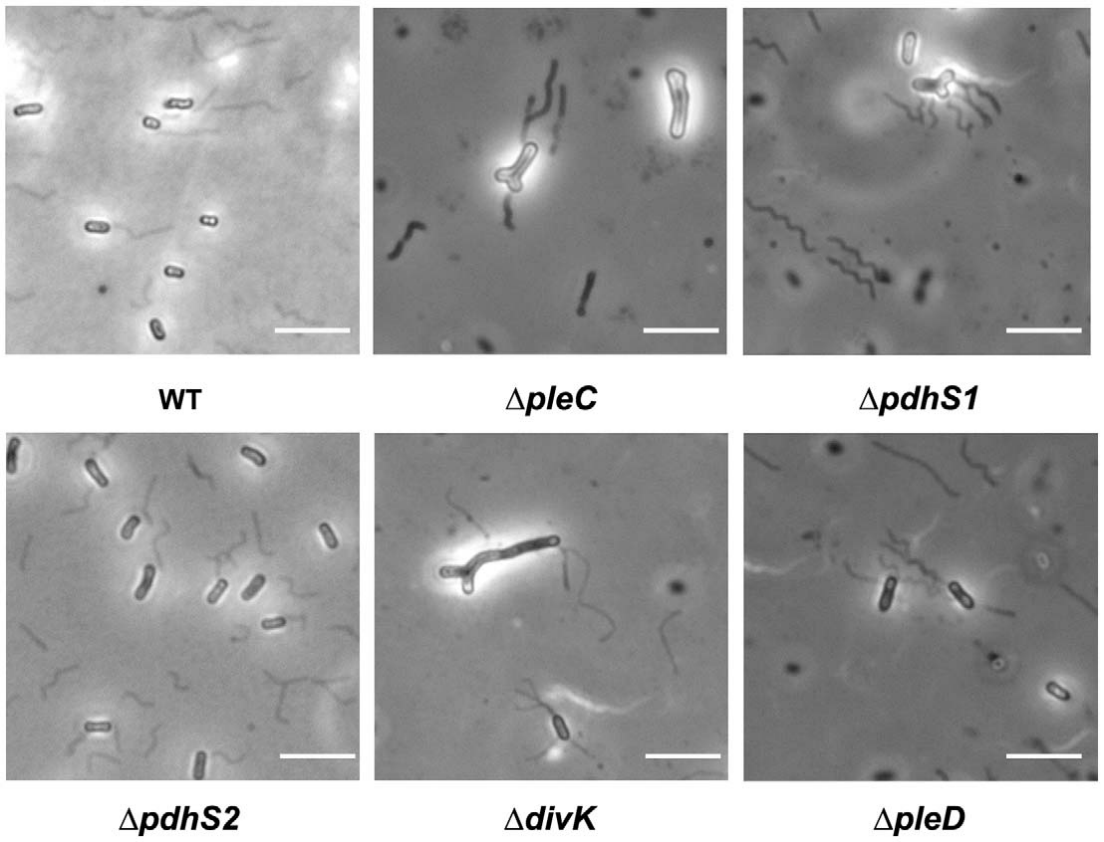
Regulation of flagella production and placement by CDD histidine kinase and response regulator mutants. Stained flagellar filaments were observed using 100X phase contrast microscopy. Scale bar = 5 µm. Note that this procedure is known to result in abundant sheared flagella (42).

In *C. crescentus* both a Δ*pleC* mutant and a Δ*divJ* mutant show defects in swimming motility, with loss of *pleC* resulting in production of an inactive flagellum [50]–[52]. We therefore examined whether the Δ*pleC*, Δ*pdhS1* and Δ*pdhS2* mutations impacted the motility of *A. tumefaciens*. All three sensor kinase mutants formed significantly smaller swim rings on 0.3% swim agar when compared with wild-type *A. tumefaciens* (Fig. 3). Complementation with plasmid-borne copies of the mutated genes corrected the motility deficiency (Table S4). Microscopic examination of both the Δ*pleC* and Δ*pdhS1* mutants in liquid culture revealed visibly swimming cells, indicating that the defect in motility on swim agar plates was likely due in part to aberrant morphology and not to a complete absence of flagellar-based motility. A qualitative crystal violet-based stain was used to verify the presence of flagella on at least a subset of cells for each strain. Wild-type *A. tumefaciens* produces a tuft of four to five flagella at one pole of the bacterium [53]. In contrast, the Δ*pleC* and Δ*pdhS1* mutants frequently localized flagella to the tip of branches, to both poles of the bacterium, or to the lateral wall of the branched bacterium (Fig. 4A). Although strongly reduced in its ability to swim in liquid culture and on 0.3% swim agar plates, flagellar positioning in the Δ*pdhS2* mutant resembled wildtype.

### Attachment and Biofilm Formation in Kinase Mutants

A complex phenotype that integrates cell growth, motility, and polar development in *A. tumefaciens* is the ability to form a biofilm [25], [28]. Biofilm formation can influence the interaction of *A. tumefaciens* with plant tissues and is tightly integrated with its physiology and morphological development. In static biofilm assays flagellar motility is required for efficient biofilm formation in *A. tumefaciens* [48]. The amount of adherent biomass that forms on plastic coverslips after 48 hours in static culture was measured for the kinase mutants. As expected, the Δ*pleC* and Δ*pdhS1* mutants were significantly reduced relative to wildtype in their ability to efficiently form a biofilm under these conditions (Fig. 3). In contrast, despite its reduced motility, the Δ*pdhS2* mutant strain was significantly elevated for biofilm formation, indicating that *pdhS2* may negatively regulate biofilm formation. Complementation with plasmid-borne copies of the mutated genes corrected the biofilm irregularities (Table S4).

**Figure 5:**
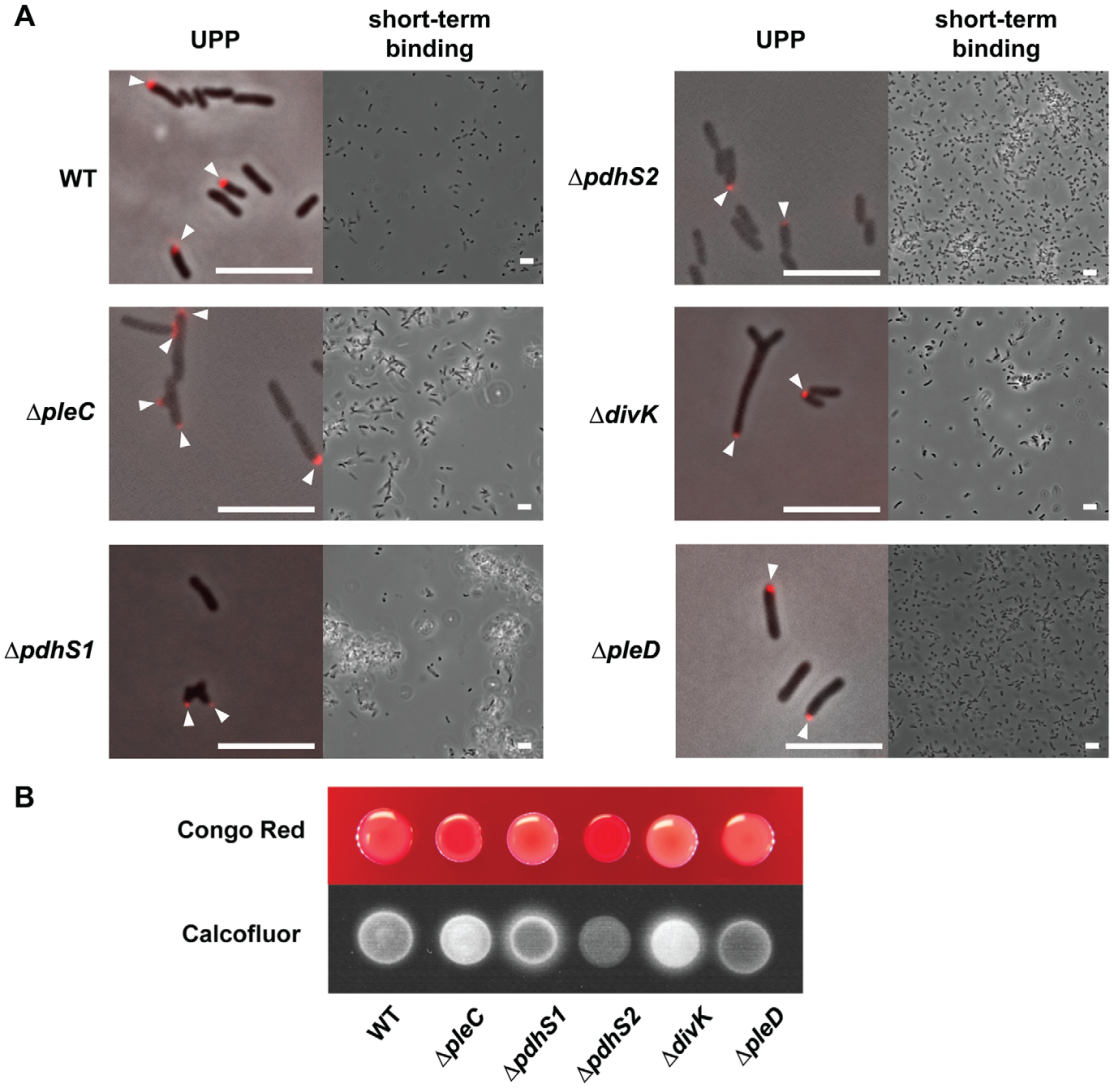
Regulation of extracellular polysaccharides and surface attachment by CDD histidine kinase and response regulator mutants. (A) Unipolar polysaccharide (UPP) production, left image, and short-term binding, right image, were monitored for each mutant. UPP production and placement was identified by the binding of Alexa Fluor 594-labeled wheat germ agglutinin to cells on 1.5% agarose pads. Arrowheads indicate UPP localization. Short-term binding was evaluated after 1 h of attachment to glass coverslips. Scale bar = 5 µm. (B) EPS production was evaluated by growth on ATGN minimal medium plates supplemented with either 100 µg·mL^−1^ Congo Red (top) or 200 µg·mL^−1^ Calcofluor White (bottom). Plates were incubated at 28° C for three days prior to imaging with standard photography (Congo Red) and using UV light exposure (280 nm, Calcofluor White).

Biofilm formation proceeds from an initial reversible binding step to an irreversible attachment mediated by a polarly localized adhesin, the unipolar polysaccharide (UPP), eventually leading to a mature biofilm [26]. The Δ*pleC*, Δ*pdhS1*, and Δ*pdhS2* mutant strains were competent for UPP elaboration as determined by lectin staining, although UPP localization was slightly altered in the Δ*pleC* and Δ*pdhS1* mutants (Fig. 5A). Normally, UPP labeling with the *N*-acetylglucosamine-specific lectin wheat germ agluttinin reveals a single, unipolar localization for the UPP [25]. Indeed, this is the case for UPP localization in the Δ*pdhS2* mutant (Fig. 5A). In the Δ*pleC* and Δ*pdhS1* mutants, however, lectin staining frequently revealed punctate staining on multiple poles of branched cells (Fig. 5A). Likewise, all three strains were competent for the initial reversible binding step as determined using a short-term (1 h) binding assay on glass coverslips (Fig. 5A). However, biofilm defects are reflected in the short-term binding assays as both the Δ*pleC* and Δ*pdhS1* mutants bound primarily as reversibly attached loose aggregates instead of the predominant irreversible polar attachment seen with the wildtype. This reversible attachment is characterized by bacterial aggregates that were not washed away during slide preparation but that did, with some frequency, detach from the coverslip and float away into the medium during microscopic examination. The Δ*pdhS2* mutant exceeded the efficiency of wild-type binding in this assay. Importantly, it was possible to identify branched cells adhering via their UPP in the Δ*pleC* and Δ*pdhS1* mutants (data not shown). Given the short incubation time for this experiment it seems likely that these cells attached to the surface after they had formed many of the observed branches, and hence branching does not preclude initial attachment.

### Extracellular Polysaccharide Production is Altered in Kinase Mutants

Among the Rhizobiales the elaboration of one or more extracellular polysaccharides (EPS) is common and often a critical determinant in establishing the host-pathogen or host-commensal relationship [54], [55]. Additionally, certain EPS molecules play important roles in adhesion, resistance to osmotic stress, and maintenance of the biofilm matrix. *A. tumefaciens* produces at least five distinct classes of EPS: cellulose (β-1,4-glucan), curdlan (β-1,3-glucan), β-1,2-glucan, succinoglycan, and the *N*-acetylglucosamine-containing unipolar polysaccharide (UPP, described above) [25], [26], [56]–[59]. To determine the effect of loss of *pleC*, *pdhS1*, or *pdhS2* on EPS production the mutants were tested on solid media containing either the polysaccharide-binding dye Congo Red, which primarily stains β-1,4-glucans such as cellulose, β-1,3-glucans, and galactoglucomannans, or Calcofluor White, which stains more generally β-linked polysaccharides, notably the complex polysaccharide succinoglycan (Fig. 5B) [47], [60], [61]. The Δ*pdhS1* mutant showed diminished binding to Congo Red when compared with the wildtype, while the Δ*pdhS2* mutant strain showed increased Congo Red binding (Fig. 5B). Reduced levels of Congo Red binding may reflect either reduced levels of cellulose or UPP production (Xu *et al*. submitted), and decreased production of either cellulose or UPP may in part explain the reduced ability to form a biofilm in the Δ*pdhS1* mutant. Conversely, increased Congo Red binding may indicate increased levels of cellulose or UPP production in the Δ*pdhS2* mutant resulting in increased biofilm formation. In contrast, the Δ*pdhS2* mutant strain was decreased for Calcofluor White fluorescence relative to wildtype, while the Δ*pdhS1* and Δ*pleC* mutant strains were increased (Fig. 5B). The Δ*pdhS1* mutant strain displayed a fluorescent halo after 3 days on Calcofluor White plates, indicative of the release of short-chain, low molecular weight EPS precursors [20]. Taken together, these results are consistent with the dysregulation of biofilm formation in these mutants. All of the mutants were capable of forming tumors when directly inoculated onto potato discs (Fig. S1). This indicates that the T-DNA transfer mechanism that underlies *A. tumefaciens* virulence is not fundamentally compromised in these mutants.

### The Response Regulators DivK and PleD Differentially Affect Development in *A. tumefaciens*

In *C. crescentus* the histidine kinases PleC and DivJ both interact with two response regulators, DivK and PleD, which regulate downstream phosphorelay components and levels of the internal second messenger, c-di-GMP, respectively (Fig. 1). *A. tumefaciens* possesses both *divK* and *pleD* homologues. Similar to *C. crescentus* the *divK* and *pleD* coding sequences are immediately adjacent to one another, likely expressed from the same transcript, and are distant from any obvious histidine kinase homologues. We were readily able to create in-frame deletion mutations in *pleD* and *divK*, the latter in contrast with *C. crescentus* and *B. abortus* in which *divK* is essential [19]. The effect of loss of either response regulator on the normal *A. tumefaciens* developmental program was evaluated for the same panel of phenotypes used for the histidine kinase mutants.

As was found for strains lacking *pleC* and *pdhS1*, the Δ*divK* mutant strain exhibited profound morphological defects, including branching, mislocalization of FtsZ, and flagellar placement, although these Δ*divK* phenotypes occurred at a lower frequency (Fig. 2 and 4A, Table S3). Doubling time was again unaffected (Table 1). Although the Δ*divK* mutant produced swim rings smaller than wildtype on motility agar, and had altered flagellar placement, the reduction in swim ring diameter was not as severe as with the kinase mutants (Fig. 3 and Fig. 4A). Motile cells were present when liquid cell suspensions were examined for the Δ*divK* mutant (data not shown). In contrast, the absence of *divK* results in a reduction in biofilm formation even more severe than that seen for either the Δ*pleC* or Δ*pdhS1* strains (Fig. 3). Complementation with a plasmid-borne copy of *divK* corrected both the swimming and biofilm deficiencies (Table S4). UPP production and short-term binding were similar to the Δ*pleC* and Δ*pdhS1* strains (Fig. 5A). Finally, the Δ*divK* mutant resembled the Δ*pdhS1* mutant in EPS production, with reduced levels of Congo Red binding and increased levels of Calcofluor White binding including a fluorescent halo (Fig. 5B). The Δ*pleD* mutant, on the other hand, demonstrated no obvious developmental defects aside from a modest increase in adherent biomass during biofilm formation (Fig. 2–5, Table S3). Significantly, EPS production, in particular the UPP, was not notably affected in the Δ*pleD* mutant (Fig. 5), in contrast to the Δ*pdhS2* mutant where its more dramatic biofilm phenotype is coincident with altered EPS production. As for the sensor kinase mutants, both the Δ*divK* and Δ*pleD* mutants were capable of forming tumors when directly inoculated onto potato discs (Fig. S1).

### Isolation and Characterization of a Spontaneous Suppressor of *ΔpleC*

**Figure 6:**
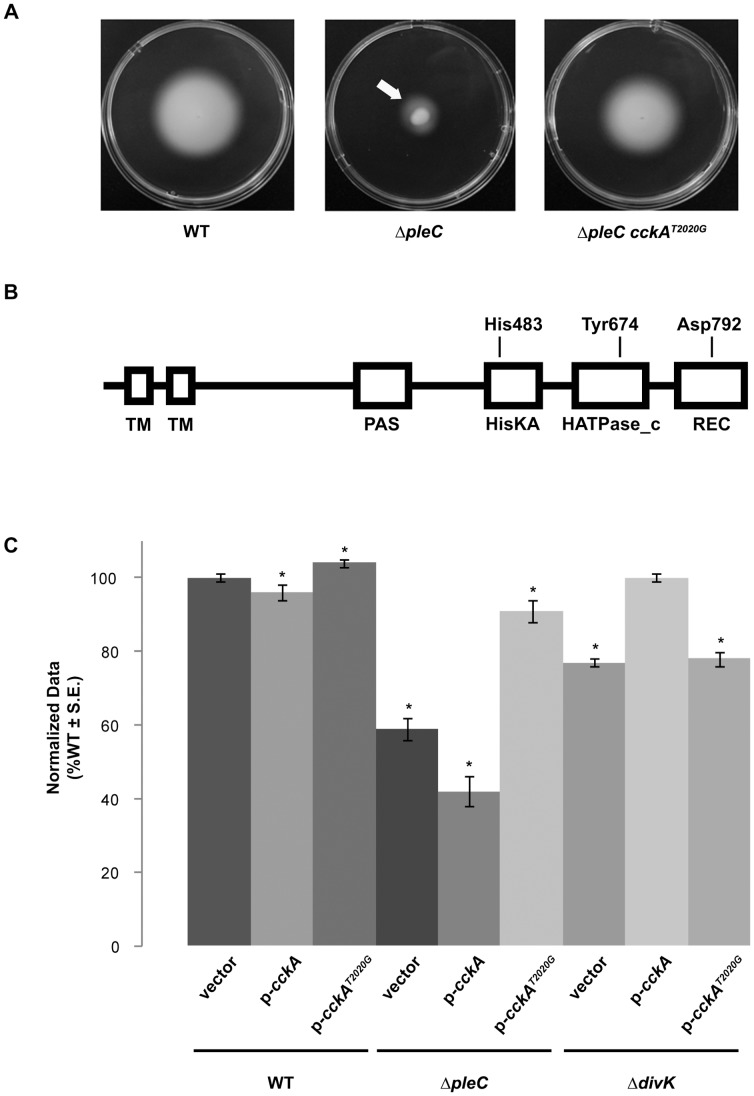
Isolation of a *cckA* mutation that suppresses Δ*pleC* mutant phenotypes. (A) Swimming motility after four days for the wild-type (WT), Δ*pleC* mutant, and Δ*pleC* suppressor strain. The suppressor strain was isolated by streak purification from the highly motile Δ*pleC* flare indicated by the white arrow. (B) Cartoon model of the CckA protein, with the N-terminus at the left and C-terminus at the right. TM = transmembrane domain, PAS = Per/ARNT/SIM domain, HisKA = histidine kinase A dimerization/phosphoacceptor domain, HATPase_c = histidine kinase-like ATPase domain, REC = signal receiver domain. Relative locations of the phosphoaccepting histidine (His483) and aspartate (Asp792) residues are indicated, as is the location of the tyrosine residue mutated in the suppressor strain (Tyr674). (C) Swim ring diameter quantification for the wild-type (WT), Δ*pleC*, and Δ*divK* mutants expressing either a wild-type copy of *cckA* or the mutant allele *cckA^T2020G^* under control of the *P_lac_* promoter, or carrying empty vector. Data are the mean of nine independent experiments (N = 9). For presentation all data were normalized to results obtained with the wild-type strain and are expressed as %WT ± S.E. Columns marked with an asterisk (*) indicate statistically significant differences between the mutant strain and the wild-type strain using Student’s *t* test (*P* ≤ 0.05).

Thus far our data implicate only components from the initial upper His-Asp phosphorelay in the CDD regulatory pathway (Fig. 1) in regulating *A. tumefaciens* division and development. The regulators in the lower pathway (*e.g.* CckA, ChpT, CtrA) cannot be deleted and are likely to be essential (data not shown). A connection between the two CDD His-Asp phosphorelays was however made during phenotypic analysis of the *A. tumefaciens* Δ*pleC* mutant. Spontaneous suppressors of the Δ*pleC* motility phenotype were frequently observed as flares on the edge of swim rings (Fig. 6A). Bacteria isolated from these flares were retested for suppression of the parental Δ*pleC* phenotypes and isolates were commonly found to restore swimming motility and biofilm formation to wild-type levels, as well as restoring normal cell morphology (Fig. 6 and data not shown). Whole genome re-sequencing of one of these suppressor mutants using Illumina technology identified a single nucleotide substitution at position 2020 of Atu1362, the *A. tumefaciens cckA* homologue. The mutation yields a singly mutated *cckA* allele, resulting in an alteration of tyrosine 674 to an aspartate residue in the CckA protein (CckA^Y674D^) (Fig. 6B). To determine whether this *cckA* allele was responsible for suppression of the Δ*pleC* motility defect we constructed an expression plasmid with either wild-type *cckA* or the CckA^Y674D^ allele expressed from an IPTG-inducible *P_lac_* promoter. These plasmids were introduced into the wild-type and Δ*pleC* mutant backgrounds, as well as the original Δ*pleC* suppressor harboring the mutant *cckA* allele. Under conditions of low (no IPTG) and high (1 mM IPTG) expression,CckA^Y674D^ *in trans* suppressed all mutant phenotypes associated with the Δ*pleC* mutant strain whereas no effect was seen in either the wild-type or the original Δ*pleC* suppressor backgrounds (Fig. 6C and data not shown). When wild-type *cckA* was strongly induced (1 mM IPTG induction) a slight inhibition of swimming motility was evident in the wild-type, Δ*pleC*, and Δ*pleC* suppressor strains. At low levels of expression (no IPTG induction) the presence of an additional copy of wild-type *cckA* had no obvious effect on swimming motility in any of the strains tested. These data suggest that when present in single copy the mutant allele of *cckA* is a dominant, gain-of-function allele. Only when the wild-type copy of *cckA* is strongly induced with high IPTG levels in combination with the native low levels of the CckA^Y674D^ variant is the suppression phenotype masked.

In *C. crescentus* a direct regulatory link between *pleC* and *cckA* is through the response regulator *divK* and its effect on *divL* [10], [62]. To determine whether the ability of CckA^Y674D^ to suppress the effects of *pleC* mutation might be due to an altered regulatory interaction with DivK we introduced a plasmid-borne copy of either the wild-type or mutant allele of *cckA*, expressed from *P_lac_*, into the Δ*divK* mutant. Under conditions of high expression (1 mM IPTG) the CckA^Y674D^ variant had no effect on swimming motility in the Δ*divK* mutant. Overexpression of the wild-type allele of *cckA*, however, restored swimming motility to normal levels in this background.

## Discussion

*A. tumefaciens* possesses the full suite of components present in the canonical CDD regulatory pathway from *C. crescentus* [17], [25]. In addition, *A. tumefaciens* contains two PdhS-type kinases, PdhS1 and PdhS2 [18]. Prior work on the *A. tumefaciens* CDD regulatory pathway suggests that it possesses a *C. crescentus*-like cell cycle and that the CcrM DNA methyltransferase, known to be regulated by CtrA in *C. crescentus*, is cell cycle-regulated and essential in this bacterium [63]. Additionally, overexpression of *ccrM* in *A. tumefaciens* was associated with branching morphologies and DNA replication errors. Our data are consistent with this and other reports indicating that the CDD regulatory pathway is functional in *A. tumefaciens* and components of this pathway regulate cell growth, division, and polar development. The aberrant branching morphologies we observe for the CDD mutants (Δ*pleC*, Δ*pdhS1*, Δ*divK*) likely reflect mislocalized initiation of the budding, asymmetric cell division cycle of *A. tumefaciens* [32]. The localization of FtsZ to these branch points supports this idea, as the cells attempt to drive septation from even these erroneous division sites. This suggests that the CDD mutants lose the ability to identify the appropriate pole for division initiation, or are not prevented from starting this process at locations away from the poles. Among recognized mechanisms for regulating FtsZ localization and activity *A. tumefaciens* possesses only the genes encoding the Min system (*minCDE*), but it remains unclear whether or how this system integrates with the CDD pathway. In *C. crescentus* CtrA represses *ftsZ* expression, thus loss of regulatory proteins in the CDD pathway are likely to affect *ftsZ* levels [64]. Control of *ftsZ* expression may therefore be unregulated in the CDD mutants and the resulting increase in FtsZ protein levels could cause formation of ectopic poles.

Our data also demonstrate that the PdhS-type kinases can impact these same growth and division phenotypes. Since the Δ*pdhS1* mutant phenocopies the Δ*pleC* and Δ*divK* mutants with respect to morphology, swimming motility, and biofilm formation, it is plausible that PdhS1 integrates directly into the CDD regulatory pathway. The intersection of PdhS2 with the CDD regulatory pathway is less certain, but the data strongly suggest that PdhS2 regulates the transition between the planktonic and sessile states of *A. tumefaciens*. Expression of either wild-type CckA or the mutant *cckA* allele, CckA^Y674D^_,_ had only modest effects on swimming motility in either the Δ*pdhS1* or Δ*pdhS2* mutant strains (data not shown), providing an important difference between the PdhS-type kinases and PleC. Experimental analysis of the Δ*pdhS1* Δ*pdhS2*, Δ*pdhS1* Δ*pleC*, and Δ*pdhs2* Δ*pleC* double mutants may help elaborate the relationships among these sensor kinase homologues.

The PdhS-type sensor kinases were first identified in *B. abortus* based on limited homology between their core catalytic domain and *C. crescentus* PleC and DivJ [18], [19]. The earlier study recognized two classes of PdhS-type kinases, differentiated by size and predicted localization. PdhS1 of *A. tumefaciens* and related homologues, including PdhS of *B. abortus* and CbrA of *S. meliloti* are large proteins (exceeding 100 kDa) with no predicted transmembrane domains. In *B. abortus* PdhS is an essential protein known to interact directly with the DivK response regulator and the FumC fumarase, and to localize at the old pole of the bacterium [19], [65]. In *S. meliloti* CbrA is non-essential and thought to regulate EPS production, motility, cell surface physiology, and symbiosis with host legumes [20], [66]. *A. tumefaciens* PdhS2 and its homologues are much smaller (∼50 kDa) and are predicted to be multi-pass integral membrane proteins. Our data show that neither PdhS1 nor PdhS2 is essential in *A. tumefaciens*, and that the downstream activities regulated by each protein affect differentiation and development of this bacterium.

The non-essentiality of *divK* and the apparent essentiality of *divJ* in *A. tumefaciens* are the most notable departures from the *C. crescentus* model for the CDD pathway. In addition, the function of PdhS1 and PdhS2 are clearly departures from this paradigm. The apparent essentiality of *divJ* is surprising in that *C. crescentus* DivJ is known to function primarily through DivK and PleD. It is unlikely that the essential activity of DivJ is mediated through PleD as the *pleD* gene, like *divK,* is non-essential. We cannot yet rule out that PleD and DivK are functionally redundant with respect to mediating the essential activity of DivJ. However, their predicted biochemical activity is decidedly distinct, with c-di-GMP production catalyzed by PleD (via its GGDEF motif), and with DivK simply encoding a response regulator receiver domain. The contrasting phenotypes observed for the Δ*pleD* and Δ*divK* mutant strains also suggest they do not function equivalently. Nonetheless, phenotypic characterization of a Δ*divK* Δ*pleD* double mutant might prove informative in understanding this module of the CDD pathway. Our preliminary data suggest that *A. tumefaciens* DivJ may have additional direct or indirect targets, apart from any interaction with PleD or DivK. One possibility is the unusual sensor kinase, DivL, described below, for which *A. tumefaciens* has a homologue (Atu0027). Also, *C. crescentus* DivJ is capable of phosphorylating CtrA *in vitro*, so perhaps DivJ directly interacts with *A. tumefaciens* CtrA *in vivo* [67]. We have recently created a *divJ* depletion strain and begun to characterize the role of *divJ* in *A. tumefaciens* development. Preliminary experiments indicate that depletion of *divJ* results in branched cells, reduced swimming motility, and increased biofilm formation. It is unclear why targets of DivJ regulation are essential, whereas the other CDD histidine kinases that affect many of the same phenotypes are not required for viability. Deletion of the *divJ* gene in the *cckA* ^Y674D^ gain of function mutant that suppresses the Δ*pleC* mutant phenotypes, has been unsuccessful despite repeated attempts, suggesting that *divJ* essentiality may be independent of its influence on the CckA-ChpT-CtrA pathway.

It is important to highlight that our results are only suggestive of possible interactions and should be interpreted conservatively. In the absence of their cognate activating kinases many response regulators may be phosphorylated by abundant small molecule phosphodonors such as acetyl phosphate or phosphoramidate [68], [69]. For many sensor kinase-response regulator pairings it is the phosphatase activity of the sensor kinase that is specific to a particular response regulator and the kinase activity of many sensor kinases is known to cross-talk with additional response regulators in a non-specific fashion [70]. Nonetheless, our findings, taken together with published data on the CDD regulatory pathway in other α-Proteobacteria, indicate robust conservation of function for the core CDD regulatory components in *A. tumefaciens*. The data are also highly suggestive that PdhS1 is directly integrated into the CDD architecture in *A. tumefaciens* and that PdhS2 plays an important role in regulating motility and attachment.

Identification of a spontaneous suppressor of the Δ*pleC* swimming motility defect as a missense mutation in the *A. tumefaciens cckA* homologue verified that the core connectivity between the two tiers of the CDD regulatory pathway is retained. Results obtained from expression of the wild-type CckA allele or singly mutated CckA^Y674D^ allele in the wild-type, Δ*pleC*, and Δ*divK* strains are consistent with the CDD model from *C. crescentus*, with DivK negatively regulating the activity of CckA and PleC primarily serving to negatively regulate DivK. This suggests that the CckA^Y674D^ variant may be less sensitive to DivK and thereby exhibits increased autophosphorylation activity. We hypothesize that the ultimate result of this increased autophosphorylation activity of CckA^Y674D^ is phosphorylation of the *A. tumefaciens* homologues of CtrA (Atu2434) and CpdR (Atu3883). If the fundamental connectivity of the CDD regulatory pathway is conserved in *A. tumefaciens*, then it is perhaps not surprising that we isolated a mutation in *cckA* as a suppressor of the Δ*pleC* swimming motility phenotype. It is likely that any mutation that results in increased activity of the downstream response regulator, CtrA, would also suppress one or more of the identified Δ*pleC* phenotypes. In *C. crescentus* mutations in both *divL* and *cckA* were identified as suppressors of multiple phenotypes in a Δ*divJ* strain [50]. The unusual histidine kinase-like protein DivL positively regulates the kinase activity of CckA and therefore the activity of CtrA [10], [71]. Both the suppressors identified in *divL* and in *cckA* resulted in increased phosphorylation, and hence activity, of CtrA. We have thus far been unable to generate deletion mutants of *cckA*, *chpT*, or *ctrA*, suggesting that these genes are essential in *A. tumefaciens*.

The CDD pathway clearly plays a crucial role in *A. tumefaciens* and its relatives, orchestrating the surprising asymmetry in their mode of growth and cellular architecture. The findings reported from this study suggest that further investigation into the architecture of the CDD regulatory pathway in *A. tumefaciens* will lead to increased insight as to how it and additional members of the α-Proteobacteria adapt to the multiple and varied environments they encounter and integrate cell growth, division, and development with changing environmental conditions, including those encountered on hosts.

## Supporting Information

Figure S1 **All five histidine kinase and response regulator mutants are virulent.** One centimeter-diameter discs of organic red potato tissue were inoculated with 50 µL of the wild-type (WT) or indicated histidine kinase or response regulator mutant strains. Inoculations were performed with three dilutions of each strain (OD_600_ = 0.6, 0.06, or 0.006). Inoculated discs were incubated at room temperature for four weeks. Images shown are from day 21 of discs inoculated with the lowest inoculum and are representative of three independent experiments each of which contained five technical replicate discs for each inoculum. Values represent mean number of tumors per disk ± S.E. Values marked with an asterisk (*) indicate statistically significant differences between the mutant strain and the wild-type strain using Student’s *t* test (*P* ≤ 0.05).
(TIF)

Table S1 **Strains and plasmids used in this study.**
(DOC)

Table S2 **Primers used in this study.**
(DOC)

Table S3 **Quantification of cellular morphology and FtsZ-GFP localization.**
(DOC)

Table S4 **Complementation of swimming motility and biofilm formation of CDD mutants.**
(DOC)
