# Supplementary figures and images for: Coordination of Division and Development Influences Complex Multicellular Behavior in Agrobacterium tumefaciens
Source: PLoS One. 2013 Feb 20;8(2):e56682. doi: 10.1371/journal.pone.0056682 (PMC3577659; doi:10.1371/journal.pone.0056682)

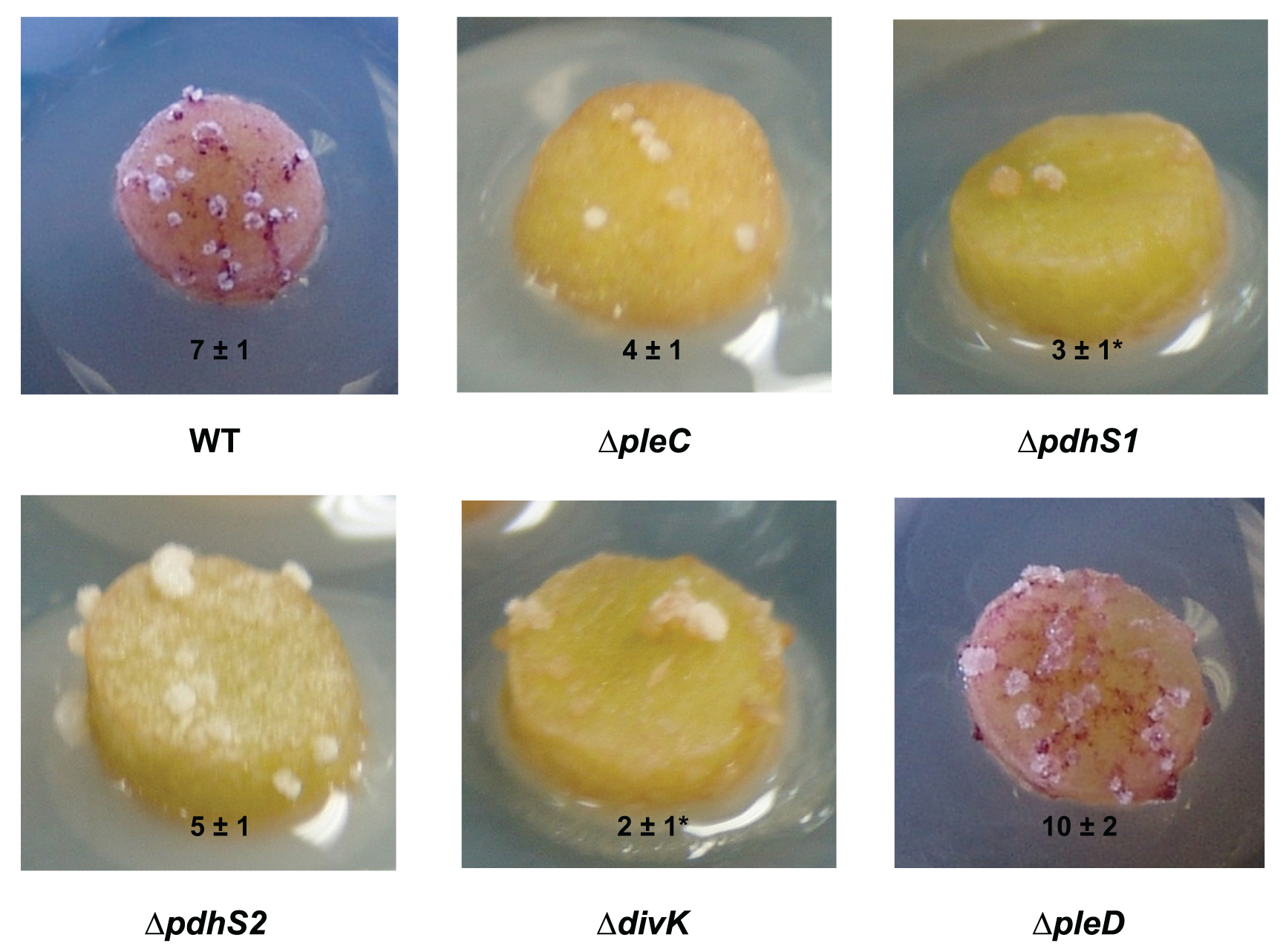

Supplement: Figure S1 — All five histidine kinase and response regulator mutants are virulent. One centimeter-diameter discs of organic red potato tissue were inoculated with 50 µL of the wild-type (WT) or indicated histidine kinase or response regulator mutant strains. Inoculations were performed with three dilutions of each strain (OD600 = 0.6, 0.06, or 0.006). Inoculated discs were incubated at room temperature for four weeks. Images shown are from day 21 of discs inoculated with the lowest inoculum and are representative of three independent experiments each of which contained five technical replicate discs for each inoculum. Values represent mean number of tumors per disk ± S.E. Values marked with an asterisk (*) indicate statistically significant differences between the mutant strain and the wild-type strain using Student’s t test (P ≤ 0.05). (TIF) [file pone.0056682.s001.tif]
