# Supplementary material for: Coordination of Division and Development Influences Complex Multicellular Behavior in Agrobacterium tumefaciens
Source: PLoS One. 2013 Feb 20;8(2):e56682. doi: 10.1371/journal.pone.0056682 (PMC3577659; doi:10.1371/journal.pone.0056682)
Supplement: Table S1 — Strains and plasmids used in this study. (DOC) [file pone.0056682.s002.doc]

| **Table S1. Strains and plasmids used in this study.** | | |
| --- | --- | --- |
| **Strain or plasmid name** | **Relevant characteristics** | **Source or reference** |
| *A. tumefaciens* strains |  |  |
| C58 | Nopaline type strain; pAtC58, pTiC58 | (Watson, 1975) |
| JEH065 | Δ*pdhS1* C58 derivative | This study |
| JEH076 | Δ*pdhS2* C58 derivative | This study |
| JEH089 | Δ*divJ* C58 derivative; depletion strain with pJEH022 | This study |
| JW2 | Δ*pleC* C58 derivative | This study |
| JW2-S | Spontaneous suppressor of JW2 | This study |
| JW8 | Δ*pleD* C58 derivative | This study |
| JW7 | Δ*divK* C58 derivative | This study |
|  |  |  |
| *E. coli* strains |  |  |
| DH5α λ *pir* | Cloning host | (Chiang, 2002) |
| S17-1 λ *pir* | Cloning host | (de Lorenzo, 1994) |
| TOP10 F’ | Cloning host | Invitrogen |
|  |  |  |
| Plasmids |  |  |
| pGEM-T Easy | PCR cloning vector; Amp^r^ | Promega |
| pJEH009 | pGEM-T Easy carrying *cckA* | This study |
| pJEH010 | pSRKGm P*_lac_*::*cckA* | This study |
| pJEH013 | pGEM-T-Easy carrying *divJ* SOE deletion fragment | This study |
| pJEH014 | pGEM-T Easy carrying *divJ* | This study |
| pJEH017 | pGEM-T Easy carrying *pdhS1* SOE deletion fragment | This study |
| pJEH019 | pNPTS138 carrying *divJ* SOE deletion fragment | This study |
| pJEH020 | pGEM-T Easy carrying *pdhS1* | This study |
| pJEH021 | pGEM-T Easy carrying *pdhS2* | This study |
| pJEH022 | pSRKGm P*_lac_*::*divJ* | This study |
| pJEH023 | pNPTS138 carrying *pdhS1* SOE deletion fragment | This study |
| pJEH025 | pSRKGm P*_lac_*::*pdhS1* | This study |
| pJEH026 | pSRKGm P*_lac_*::*pdhS2* | This study |
| pJEH029 | pGEM-T Easy carrying *cckA^T2020G^* | This study |
| pJEH030 | pSRKGm P*_lac_*::*cckA^T2020G^* | This study |
| pJEH036 | pGEM-T Easy carrying *pdhS2* SOE deletion fragment | This study |
| pJEH040 | pNPTS138 carrying *pdhS2* SOE deletion fragment | This study |
| pJW2 | pGEM-T Easy carrying *pleC* SOE deletion fragment | This study |
| pJW10 | pGEM-T Easy carrying *pleD* SOE deletion fragment | This study |
| pJW11 | pGEM-T Easy carrying *pleD* | This study |
| pJW23 | pGEM-T Easy carrying *gfp*mut3 | This study |
| pJW27 | pGEM-T Easy carrying *pleC* | This study |
| pJW41 | pGEM-T Easy carrying *divK* SOE deletion fragment | This study |
| pJW42 | pGEM-T Easy carrying *divK* | This study |
| pJW102 | pNPTS138 carrying *pleC* SOE deletion fragment | This study |
| pJW109 | pNPTS138 carrying *pleD* SOE deletion fragment | This study |
| pJW110 | pSRKKm P*_lac_*::*pleD* | This study |
| pJW123 | pSRKKm P*_lac_*::*gfp*mut3 | This study |
| pJW127 | pSRKKm P*_lac_*::*pleC* | This study |
| pJW141 | pNPTS138 carrying *divK* SOE deletion fragment | This study |
| pJW142 | pSRKKm P*_lac_*::*divK* | This study |
| pJW164G | pSRKKm P*lac*::*ftsZ*::*gfpmut3* | (Brown, 2012) |
| pJZ383 | P*_tac_*::*gfp*mut3 Sp^r^; pVS replicon | (Ramey, 2004) |
| pNPTS138 | colE1 origin; *sacB*; Km^r^ | gift of M. Alley |
| pSRKGm | Broad host range vector containing P*_lac_*; *lacI^q^*; *lacZα^+^*; Gm^r^ | (Khan, 2008) |
| pSRKKm | Broad host range vector containing P*_lac_*; *lacI^q^*; *lacZα^+^*; Km^r^ | (Khan, 2008) |
