# Supplementary material for: Coordination of Division and Development Influences Complex Multicellular Behavior in Agrobacterium tumefaciens
Source: PLoS One. 2013 Feb 20;8(2):e56682. doi: 10.1371/journal.pone.0056682 (PMC3577659; doi:10.1371/journal.pone.0056682)
Supplement: Table S2 — Primers used in this study. (DOC) [file pone.0056682.s003.doc]

| **Table S2. Primers used in this study.** | | |
| --- | --- | --- |
| **Primer** | **Sequence** | **Use** |
| divK1 | actagtGTCAATACACACCTGAATCCAG | P1 for *divK* SOE deletion fragment |
| divK2 | *AAGCTTGGTACCGAATTC*TATCATGACCTGCTTGGGCATGGT | P2 for *divK* SOE deletion fragment |
| divK3 | *GAATTCGGTACCAAGCTT*AAGACCTATCTTGGCGATGCGTAA | P3 for *divK* SOE deletion fragment |
| divK4 | gcatgcCAGCATCTCCTCGATGCCGATCTG | P4 for *divK* SOE deletion fragment |
| divK5 | catatgCCCAAGCAGGTCATGATA | *divK* cloning |
| divK6 | tctagaTTACGCATCGCCAAGATAGGT | *divK* cloning |
| divK7 | CAGAACGTAGCCAACCAT | External sequencing primer for *divK* locus |
| divK8 | ATGCGTTCCTGCGAGCTG | External sequencing primer for *divK* locus |
| JEH26 | gaagaacatatgACACGGGTGCGCGAGACAA | *cckA* cloning |
| JEH27 | gaagaagctagcTCACTCCTTGTCGTCGACGA | *cckA* cloning |
| JEH42 | actagtGGACAGCGCCACGAGCACCA | P1 for *divJ* SOE deletion fragment |
| JEH43 | TTAGGCGATTTTCGCTTTCGC*TGCGACCGCTTTTTCTCTCAT* | P2 for *divJ* SOE deletion fragment |
| JEH44 | *ATGAGAGAAAAAGCGGTCGCA*GCGAAAGCGAAAATCGCCTAA | P3 for *divJ* SOE deletion fragment |
| JEH45 | gcatgcGCAACGGTCGCGCCATCGA | P4 for *divJ* SOE deletion fragment |
| JEH46 | gaagaacatatgATGAGAGAAAAAGCGGTCGCA | *divJ* cloning |
| JEH52 | gaagaagctagcTTAGGCGATTTTCGCTTTCGCGG | *divJ* cloning |
| JEH55 | actagtGCGCAGACGATCCCGAAGAA | P1 for *pdhS1* SOE deletion fragment |
| JEH56 | AGAATAGGTTTCGACATGCCC*AACTAAGGCCATGCGGGCGTA* | P2 for *pdhS1* SOE deletion fragment |
| JEH57 | *TACGCCCGCATGGCCTTAGTT*GGGCATGTCGAAACCTATTCT | P3 for *pdhS1* SOE deletion fragment |
| JEH58 | gcatgcGAACAGGTGTTAATAGGGAGAGAA | P4 for *pdhS1* SOE deletion fragment |
| JEH59 | gaagaatctagaATGCCCGCCGTCCAATA | *pdhS1* cloning |
| JEH60 | gaagaactcgagTTAGTTGGCAAGAACCCGC | *pdhS1* cloning |
| JEH62 | *GAGCTTTGGTGCGCTCTAGGC*ACTCATTGTTACAACGCAGGT | P2 for *pdhS2* SOE deletion fragment |
| JEH63 | ACCTGCGTTGTAACAATGAGT*GCCTAGAGCGCACCAAAGCTC* | P3 for *pdhS2* SOE deletion fragment |
| JEH64 | gcatgcGGTGAAGGCACAGGTGCA | P4 for *pdhS2* SOE deletion fragment |
| JEH65 | gaagaacatatgAGTAAAAGCGTCAGCA | *pdhS2* cloning |
| JEH66 | gaagaagctagcCTAGGCGAAAGACCGCCG | *pdhS2* cloning |
| JEH91 | GCGCCCTTGACGCAGACA | External sequencing primer for *divJ* locus |
| JEH92 | GGCATTGGCCGAAGTGTTGA | External sequencing primer for *divJ* locus |
| JEH100 | GCTCTGTTGAAGGCGGCCAA | External sequencing primer for *pdhS2* locus |
| JEH101 | GCGAACGGCACTCTGCA | External sequencing primer for *pdhS1* locus |
| JEH102 | GCGCAGGCGATTTTAGCCAA | External sequencing primer for *pdhS1* locus |
| JEH105 | GAGACGACAGCGGGCGATCT | P1 for *pdhS2* SOE deletion fragment |
| JEH113 | GCCGGTTTCATGCACACGCA | External sequencing primer for *pdhS2* locus |
| OLJ131 | AATGATACGGCGACCACCGAGATCTACACTCTTTCCCTACACGACGCTCT**T**C**C**GATCT | Adapter for construction of paired-end library |
| OLJ137* | GATCGGAAGAGCGGTTCAGCAGGAATGCCGAGACCGATCTCGTATGCCGTCTTCTGCTTG | Adapter for construction of paired-end library |
| OLJ139 | AATGATACGGCGACCACCGAGATCTACACTCTTTCCCTACACGA | Amplifying primer for construction of paired-end library |
| OLJ140 | CAAGCAGAAGACGGCATACGAGATCGGTCTCGGCATTCCTGCTGAAC | Amplifying primer for construction of paired-end library |
| pleC1 | tctagaACATGTCGAGGACCAGCGC | P1 for *pleC* SOE deletion fragment |
| pleC2 | *AAGCTTGGTACCGAATTC*CACGTTCGTCATTGTCCCGCCTTT | P2 for *pleC* SOE deletion fragment |
| pleC3 | *GAATTCGGTACCAAGCTT*GGGACGGTGCATTGAAGCTTGGTG | P3 for *pleC* SOE deletion fragment |
| pleC4 | gcatgcATCGACCTCGAATTCATC | P4 for *pleC* SOE deletion fragment |
| pleC5 | GATCAGAAAGGAATTGCAGATGAC | External sequencing primer for *pleC* locus |
| pleC6 | ACTGGGATTTCAAGCTCATCAATG | External sequencing primer for *pleC* locus |
| pleC10 | ctcgagATGCACCGTCCCACACACCCCTGC | *pleC* cloning |
| pleC11 | tctagaCTGAAACAGGAAACAGCTATGACGAACGTGCGGCGGGCGACT | *pleC* cloning |
| pleD1 | actagtCTAGCCTTGCGCAACGAA | P1 for *pleD* SOE deletion fragment |
| pleD2 | *AAGCTTGGTACCGAATTC*AACCAGAACTCTCGCCGTCATAAC | P2 for *pleD* SOE deletion fragment |
| pleD3 | *GAATTCGGTACCAAGCTT*CTTTACGAGGCGAAACATGCCGGA | P3 for *pleD* SOE deletion fragment |
| pleD4 | gcatgcGATTCGGACATCGTCAG | P4 for *pleD* SOE deletion fragment |
| pleD5 | GTCTCGCGCAGCATTTCTTC | External sequencing primer for *pleD* locus |
| pleD6 | GTGATTACGGCACGAGGAAC | External sequencing primer for *pleD* locus |
| pleD7 | catatgACGGCGAGAGTTCTG | *pleD* cloning |
| pleD8 | ggatccAACAGTCGTGGTTAATTG | *pleD* cloning |
| pUC/M13F | CGCCAGGGTTTTCCCAGTCACGAC | pGEM-T Easy and pNPTS138 sequencing |
| pUC/M13R | TCACACAGGAAACAGCTATGAC | pGEM-T Easy and pNPTS138 sequencing |

Underlining highlights features engineered for optimized expression. Lowercase indicates engineered restriction sites. Italics indicates overlapping sequences for respective P2/P3 primers used to generate SOE deletion fragments. Bold indicates locked nucleic acids. * OLJ137 is phosphorylated on the 5’ end.
