# Supplementary material for: Coordination of Division and Development Influences Complex Multicellular Behavior in Agrobacterium tumefaciens
Source: PLoS One. 2013 Feb 20;8(2):e56682. doi: 10.1371/journal.pone.0056682 (PMC3577659; doi:10.1371/journal.pone.0056682)
Supplement: Table S3 — Quantification of cellular morphology and FtsZ-GFP localization. (DOC) [file pone.0056682.s004.doc]

**Table S3. Quantification of cellular morphology and FtsZ-GFP localization.**

| **Strain** | **Morphology**  **(% abnormal ± S.D.)** | **FtsZ-GFP Localization (% aberrant ± S.D.)** |
| --- | --- | --- |
| WT | <1 ^a^ | 13 ± 10 |
| Δ*pleC* | 33 ± 8 ^b^ | 46 ± 15 ^c^ |
| Δ*pdhS1* | 43 ± 7 ^b^ | 39 ± 11 ^c^ |
| Δ*pdhS2* | 4 ± 2 ^c^ | 13 ± 10 |
| Δ*divK* | 28 ± 5 ^b^ | 22 ± 5 |
| Δ*pleD* | 4 ± 3 | 15 ± 6 |

^a^ 0/156 cells observed displayed abnormal morphology.

^b^ *P* ≤ 0.005 compared to the wild-type strain using Student’s *t* test.

^c^ *P* ≤ 0.05 compared to the wild-type strain using Student’s *t* test.
