# Supplementary material for: Coordination of Division and Development Influences Complex Multicellular Behavior in Agrobacterium tumefaciens
Source: PLoS One. 2013 Feb 20;8(2):e56682. doi: 10.1371/journal.pone.0056682 (PMC3577659; doi:10.1371/journal.pone.0056682)
Supplement: Table S4 — Complementation of swimming motility and biofilm formation of CDD mutants. (DOC) [file pone.0056682.s005.doc]

**Table S4. Complementation of swimming motility and biofilm formation of CDD mutants.**

| **Strain** | **Adherent Biomass (% WT ± S.E.)** | **Swim Ring Diameter (% WT ± S.E.)** |
| --- | --- | --- |
| Δ*pleC* p-*pleC* | 121 ± 4 ^a, b^ | 99 ± 2 ^b^ |
| Δ*pdhS1* p-*pdhS1* | 90 ± 4 ^a, b^ | 96 ± 1 ^a, b^ |
| Δ*pdhS2* p-*pdhS2* | 105 ± 6 ^b^ | 74 ± 1 ^a, b^ |
| Δ*divK* p-*divK* | 81 ± 5 ^a, b^ | 98 ± 2 ^b^ |
| Δ*pleD* p-*pleD* | 822 ± 29 ^a, b^ | 95 ± 1 ^a^ |

^a^ *P* ≤ 0.05 compared to wild-type strain with vector only.

^b^ *P* ≤ 0.05 compared to identical strain background with vector only.
